# Characterization of Ascites-Derived Ovarian Tumor Cells from Spontaneously Occurring Ovarian Tumors of the Chicken: Evidence for E-Cadherin Upregulation

**DOI:** 10.1371/journal.pone.0057582

**Published:** 2013-02-27

**Authors:** Anupama Tiwari, Jill A. Hadley, Gilbert L. Hendricks, Robert G. Elkin, Timothy Cooper, Ramesh Ramachandran

**Affiliations:** 1 Department of Animal Science, Center for Reproductive Biology and Health, The Pennsylvania State University, University Park, Pennsylvania, United States of America; 2 Department of Comparative Medicine, Penn State College of Medicine, Penn State Hershey Medical Center, Hershey, Pennsylvania, United States of America; University of Alabama at Birmingham, United States of America

## Abstract

Ovarian cancer, a highly metastatic disease, is the fifth leading cause of cancer-related deaths in women. Chickens are widely used as a model for human ovarian cancer as they spontaneously develop epithelial ovarian tumors similar to humans. The cellular and molecular biology of chicken ovarian cancer (COVCAR) cells, however, have not been studied. Our objectives were to culture COVCAR cells and to characterize their invasiveness and expression of genes and proteins associated with ovarian cancer. COVCAR cell lines (n = 13) were successfully maintained in culture for up to19 passages, cryopreserved and found to be viable upon thawing and replating. E-cadherin, cytokeratin and α-smooth muscle actin were localized in COVCAR cells by immunostaining. COVCAR cells were found to be invasive in extracellular matrix and exhibited anchorage-independent growth forming colonies, acini and tube-like structures in soft agar. Using RT-PCR, COVCAR cells were found to express E-cadherin, N-cadherin, cytokeratin, vimentin, mesothelin, EpCAM, steroidogenic enzymes/proteins, inhibin subunits-α, βA, βB, anti-müllerian hormone, estrogen receptor [ER]-α, ER-β, progesterone receptor, androgen receptor, and activin receptors. Quantitative PCR analysis revealed greater N-cadherin, vimentin, and VEGF mRNA levels and lesser cytokeratin mRNA levels in COVCAR cells as compared with normal ovarian surface epithelial (NOSE) cells, which was suggestive of epithelial-mesenchymal transformation. Western blotting analyses revealed significantly greater E-cadherin levels in COVCAR cell lines compared with NOSE cells. Furthermore, cancerous ovaries and COVCAR cell lines expressed higher levels of an E-cadherin cleavage product when compared to normal ovaries and NOSE cells, respectively. Cancerous ovaries were found to express significantly higher ovalbumin levels whereas COVCAR cell lines did not express ovalbumin thus suggesting that the latter did not originate from oviduct. Taken together, COVCAR cell lines are likely to improve our understanding of the cellular and molecular biology of ovarian tumors and its metastasis.

## Introduction

Ovarian cancer is the fifth leading cause of cancer-related deaths in women [1]–[3]. According to an estimate by the National Cancer Institute, the number of new cases of ovarian cancer diagnosed in women will be 22,880 and deaths caused by ovarian cancer will be 15,500 by the end of 2012 [4]. Although surgical and chemotherapeutic interventions have improved 5- year survival rate, the cure rate of all stages of ovarian cancer is less than 40% [5]. Ovarian cancer is often diagnosed at an advanced stage of disease progression when metastasis of tumor cells has already begun [6]. Ovarian cancer prevention, therefore, assumes greater importance in order to reduce the incidence of this deadly neoplasm.

Animal models that develop epithelial ovarian cancer spontaneously are important for cancer prevention studies and to understand early events in ovarian tumorigenesis. In this regard, the domestic chicken (*Gallus domesticus*) is the most appropriate animal model for human epithelial ovarian cancer [7]–[10]. Laying hens develop epithelial ovarian tumors spontaneously with similar etiology, undergo similar disease progression [11], and exhibit a high rate of ovarian cancer incidence (25–40% between 2 and 4 years of age). Hens ovulate almost daily and have 450 ovulations or more within 2 years of age. Such incessant ovulation is likely to contribute to the high rate of ovarian cancer incidence by possibly causing oxidative DNA damages in ovarian surface epithelial cells (OSE; [12]).

Recently, several ovarian cancer prevention studies using the chicken model have been reported. Administration of medroxyprogesterone acetate for 16 months in chickens led to a reduction in the incidence of reproductive tract adenocarcinoma [10]. A low-calorie diet over a 2 year-period resulted in fewer ovulations and also reduced the incidence of ovarian adenocarcinoma and p53 tumor suppressor gene mutations [13]. Supplementing the hen’s diet with 0.1% aspirin for 1 year resulted in fewer cases of advanced stage ovarian carcinoma [14]. Similarly, feeding diets enriched with flaxseed meal resulted in the reduction of the severity of ovarian adenocarcinoma [15].

Although the chicken ovarian cancer model has been gaining importance in the last 10 years, our knowledge of the cellular and molecular biology of chicken ovarian cancer (COVCAR) cells remains very limited. Currently, there are no immortalized or primary COVCAR cell lines available. In previous attempts, chicken OSE cells were found to express cytokeratin and vimentin when maintained in culture for 10 days but failed to attach or proliferate following trypsinization or non-enzymatic dissociation procedures [16]. A recent report, however, describes harvesting of OSE cells from normal and cancerous chicken ovarian tissue and maintaining them in culture for 2–3 passages for determination of CpG methylation status of SERPINB11 gene [17]. Tumor cells dispersed by enzymatic digestion from chicken ovary were cultured for 6 days to determine secretion of CA125 antigen [18]. Ascites development is one of the features of advanced stages of ovarian cancer in chickens and ascites-derived cells were maintained in short-term culture for determination of vascular endothelial growth factor (VEGF) expression [19]. Other important properties of chicken ovarian tumor cells, in particular, including morphological characteristics, invasiveness, and expression of genes/proteins related to ovarian tumor and metastasis remain unknown. Such information is essential for understanding the development of ovarian tumor and its metastasis in chickens. Therefore, the primary objectives of this study were to establish that COVCAR cells can be grown *in vitro* and to determine their invasive properties and anchorage-independent growth. Additionally, we sought to elucidate expression of various ovarian tumor-related genes and proteins in COVCAR cells.

## Results

### Ovarian Tumor Histology and Ascites

**Figure 1:**
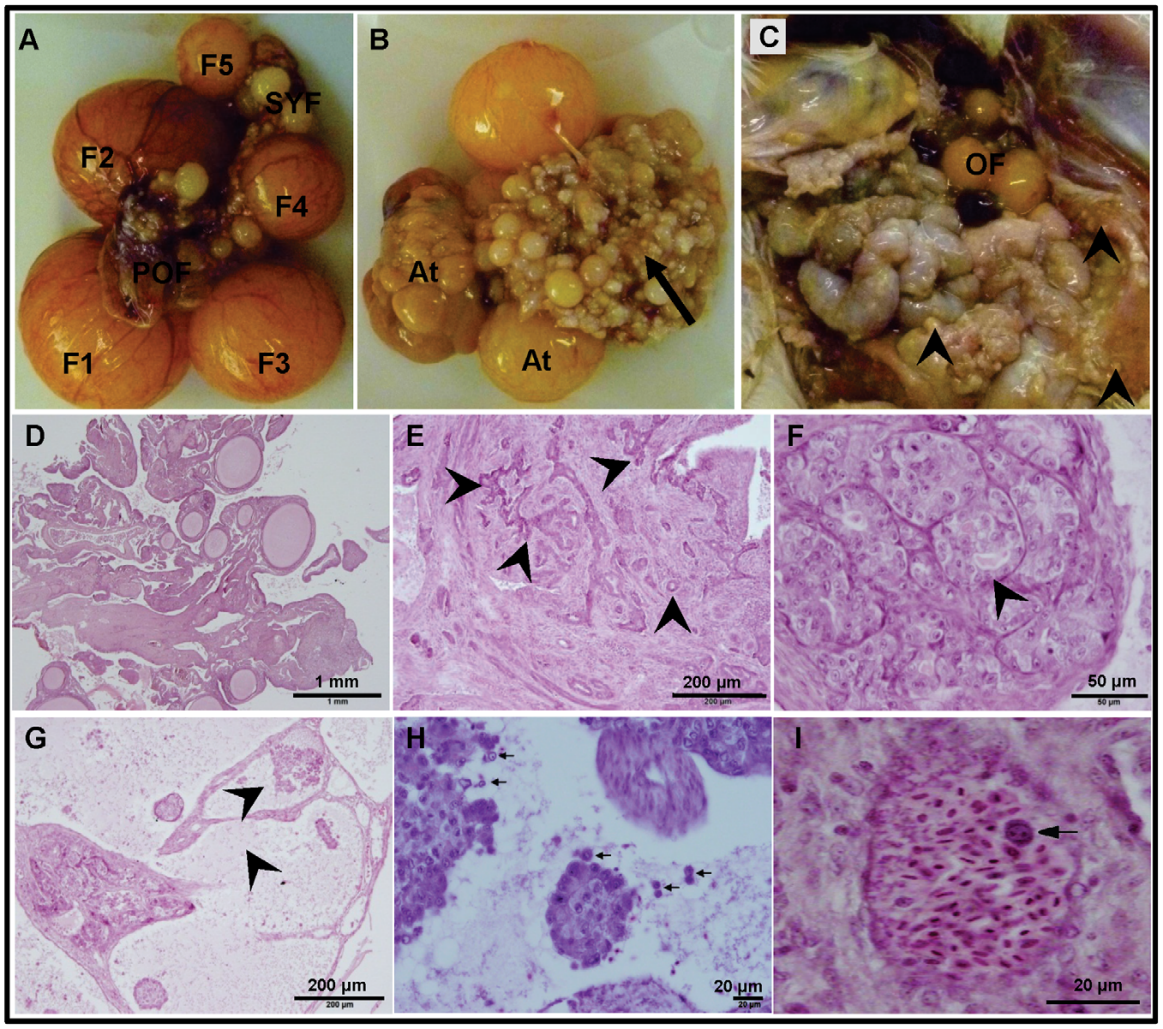
Gross morphology and histology of normal and cancerous chicken ovaries. A. Normal ovary containing a hierarchy of pre-ovulatory follicles (F1–5), pre-hierarchical follicles (SYF), and post-ovulatory follicle (POF). **B.** Tumor mass (*arrow*) and a few atretic pre-ovulatory follicles (At). **C.** Abdominal viscera showing tumors nodules on intestinal wall and peritoneum (*arrow heads*) and a few ovarian follicles (OF). **D-I.** Photomicrographs of hematoxylin and eosin stained ovarian tissue sections. **D.** Normal ovarian stroma containing several undifferentiated follicles. **E–I**. Cancerous ovarian tissue section showing coarse fibrous stroma containing several acini and ducts (*arrows heads*; **E)**, multiple cysts comprised of anaplastic cells (**F**), cystic spaces containing papillary projections of neoplastic cells (**G**), dyscohesive cells **(H),** and tumor-cell emboli in arterial lumen (**I**).

One of the objectives of this study was to characterize cells derived from ascites from chickens that had developed ovarian carcinoma. Thirty-three of the 50 hens that were euthanized were found to be normal (cancer-free) and their ovaries contained a typical hierarchy of 4–6 pre-ovulatory follicles and several pre-hierarchical follicles (Fig. 1 A). Histologically, the normal ovarian stroma (Fig. 1 D) contained several smaller follicles lined by a layer of thecal cells. In contrast, 17 of the hens exhibited Stage III or Stage IV [11] of ovarian carcinoma which was characterized by the presence of ascites and visible tumor mass on the ovary and other visceral organs and peritoneum (Table 1). The tumor mass on the ovary had the characteristic papillary outgrowths of varying sizes (Fig. 1B). Numerous tumor nodules were present on the walls of the intestine, peritoneum, and mesentery (Fig. 1C). The tumor mass in oviduct, if present, mainly involved the infundibular and magnum regions and resembled the ovarian tumor mass (data not shown). Upon histopathological analyses, the ovarian tumor masses were found to be endometriod type of adenocarcinoma and/or anaplastic carcinoma. The coarse fibrous stroma of the ovary was multifocally infiltrated and expanded by moderately to poorly differentiated epithelial cells forming acini and ducts (Fig. 1 E) as well as solid nests and cords. Some ducts/acini were concentrically surrounded by a looser stroma with a higher density of plump spindloid cells. In some areas, neoplastic cells had a spindloid appearance possibly due to epithelial-mesenchymal transformation (data not shown). In several animals, the ovarian tumor contained several back to back solid nests exhibiting anaplasia (Fig. 1F). In multiple ovaries, there were cystic spaces containing papillary projections of neoplastic cells while the cells within these spaces were frequently dyscohesive (Fig. 1 G and H). Interestingly, intravascular tumor cell-emboli were also observed (Fig. 1 I) that is indicative of ovarian cancer cell invasion into vascular system and metastasis.

**Table 1:** Characteristics of chickens with Stage III or IV ovarian tumor and associated chicken ovarian cancer (COVCAR) cell lines.

COVCAR cell line/Animal ID	Age (years)	Ascites volume (ml)	Color	Metastasis	Maximum number of passages
C3	3	165	straw	peritoneum, intestine, oviduct	5
C4	3	50	greenish yellow	peritoneum, intestine	15
C5	3	40	straw	oviduct, intestine	12
C6	3	430	yellow	intestine	8
C7	3	470	greenish yellow	oviduct, intestine	8
C8	4	700	straw	oviduct, intestine, peritoneum, liver	3
C9	4	750	straw	oviduct, intestine, peritoneum, pancreas	7
C10	4	30	red; mixedwith blood	oviduct	cells could notbe isolated
C11	4	615	straw	oviduct, intestine, peritoneum	19
C12	4	150	straw	oviduct, intestine	7
C13	3	190	straw	none detected	5
C14	3	320	straw	peritoneum, intestine, fibrosedduodenum	cells did not grow
C15	3	160	straw	peritoneum, intestine, oviduct	5
C16	3	50	red; mixed with blood	Intestine, pancreas	3
C17	4	30	yellow; mixed with yolk	oviduct, intestine	cells could notbe isolated
C18	4	20	yellow; mixed with yolk	intestines, peritoneum, liver	cells could notbe isolated
C19	3	800	straw	oviduct	16

Most of the animals that had ovarian tumors were not ovulating for at least 30 days and were asymptomatic except for the distended appearance of the abdomen in some animals. The ascites volume exceeded 500 ml in several animals (Table 1) while the ascites color was generally straw-colored in most of the animals but was mixed with blood or yolk possibly arising from rupturing ovarian follicles (C10, C16, C17, and C18; Table 1). Other conditions associated with ascites such as liver damage, heart failure, and avian tuberculosis were ruled out based on the history, absence of clinical signs, and normal appearance of associated organs by gross examination. Cells present in the ascites were recoverable from all of the animals except from a few animals (C10, C17, and C18) in which the ascites was mixed with excessive blood or yolk. Out of the 17 chickens that had ovarian tumor and ascites, we harvested cells from 13 animals, cultured, and cryopreserved them (Table 1). There was no ascites found in normal hens that were ovulating regularly (data not shown).

### COVCAR Cell Lines Grow Robustly *in vitro*

**Figure 2:**
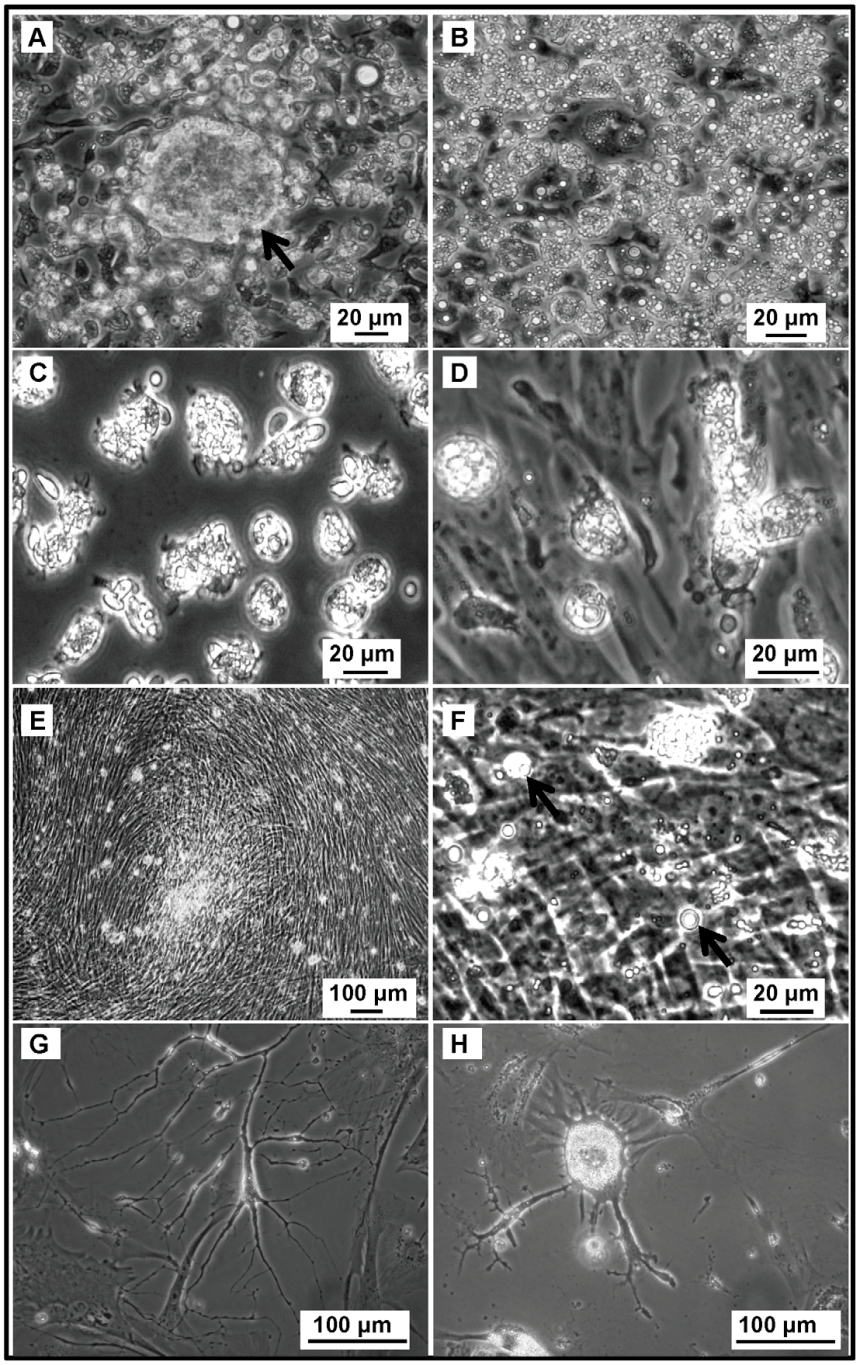
Ascites-derived chicken ovarian cancer (COVCAR) cells in culture. COVCAR cells were harvested from ascites and cultured as described in *Materials and Methods* section. Notice several translucent vesicles in the cytoplasm and spherical shape of the cells (**A, B, C,** and **D**). A few large spheroid mass of COVCAR cells were noticed as shown in **A** (arrow). Many cells had papilla-like or microvilli-like projections on the cell surface (**C**). Notice fibroelastic transformation of spherical COVCAR cells (**D**)**. E-F.** Multiple layers of fully confluent COVCAR cells appearing as an interwoven mat. A few floating cells as shown in **F** (*arrows*) displayed translucent vesicles in the cytoplasm. **G**. COVCAR cells appearing as a network of tube-like structure. **H.** A few senescent cells showing cellular hypertrophy and stellate appearance.

COVCAR cells derived from ascites of each animal that was found to have ovarian cancer (Table 1) were maintained and propagated by routine passaging until the cells reached senescence. The morphology and behavior of the COVCAR cell lines from all the 13 animals were highly similar to each other with the exception of the number of times each COVCAR cell line could be passaged before reaching senescence (ranging between 3 and 19 passages; Table 1). Possible factors contributing to the differences in the viability of COVCAR cells upon repeated passaging include ability of ovarian tumor cells to adapt to cell culture media, attachment to plastic surface, and to enzymatic digestion during passaging. In addition, the number of malignant tumor cells recovered from ascites may have also affected their viability upon repeated passaging. Immediately after recovery from ascites, COVCAR cells appeared spherical and contained several translucent vesicles (Fig. 2 A, B, C, D). A few large spheroid masses of COVCAR cells were noticed in the first 24 h of culture (Fig. 2 A). Some of the cells showed papilla-like or microvilli-like projections (Fig. 2 C) on the cell surface immediately after plating while the cell surface appeared relatively smoother in some cells (Fig. 2 D). Following the initial plating, varying numbers of erythrocytes were detected in the culture flask, but they did not attach and were readily removed by washing or during media exchange. Cell doubling time was approximately 48 h in earlier passages and required frequent media change in COVCAR cell lines. At 100% confluence, cells appeared as an interwoven mat with no intercellular spaces (Fig. 2 E, F). COVCAR cells appeared to have lost polarity and contact-inhibition as they continued to proliferate even after reaching 100% confluence leading to the formation of multi-layered stacks of adherent cells (Fig. 2 F) with numerous cells found floating in the culture media. Such floating cells were found to be viable by replating them after recovery from the over-confluent flasks. During initial plating, COVCAR cells readily proliferated forming a uniform layer with cobblestone appearance (data not shown). All the COVCAR cell lines were easily detached and passaged following enzymatic digestion. Successful propagation of the cell lines, however, required replating of cells at a high density to allow cell-cell contact. During later passages, however, COVCAR cells did not maintain a typical epithelial arrangement but developed as a layer of spindle to fibroelastic cells. Many of the COVCAR cells were multi-nucleated suggesting either high proliferation rate and/or defects in cytokinesis (data not shown) while some of the COVCAR cells appeared as a network of tube-like structures (Fig. 2 G). All COVCAR cell lines eventually reached senescence characterized by cellular hypertrophy, stellate appearance (Fig. 2 H), and slow proliferation rate. Cryopreserved COVCAR cells obtained from earlier passages were viable and exhibited robust growth upon thawing and subsequent culturing. In contrast to COVCAR cell lines, NOSE cells obtained from the ovarian follicular surface were relatively slower to attach (>6 days) and exhibited slow growth. NOSE cells were detachable from culture flasks by enzymatic digestion and withstood subsequent 1–3 passages before becoming senescent.

### COVCAR Cell Lines Display Anchorage-independent Growth *in vitro*

**Figure 3:**
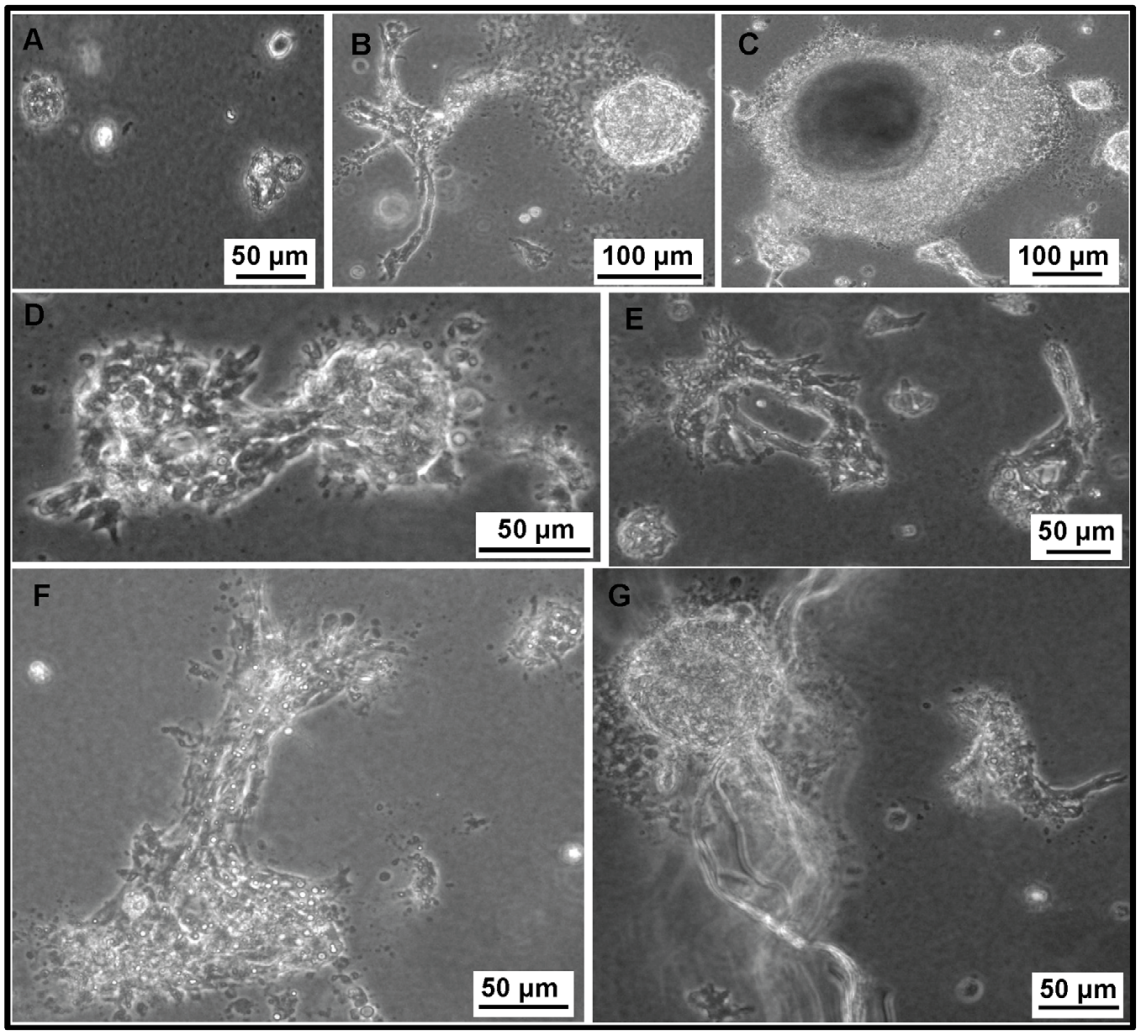
Anchorage-independent growth of chicken ovarian cancer (COVCAR) and normal ovarian surface epithelial (NOSE) cells. COVCAR and NOSE cells were cultured in semi-solid media for 4 weeks as described in *Materials and Methods* section. **A**. NOSE cells remaining as single cells showing no detectable changes in morphology. **B.** COVCAR cells on day 6 of culture showing numerous projections on the surface and forming solid ball-like structure. **C-D**. Multiple colonies and a large sphere-like structure of COVCAR cells on day 11 of culture. **E.** Two acini composed of COVCAR cells on day 13 of culture. **F.** Proliferating COVCAR cells invading through agarose. **G**. A large sphere of COVCAR cells and several tube-like structures. Scale bars = 100 µm.

Anchorage-independent growth of representative COVCAR cell lines (C5, C6, C11, C19) and NOSE cells was studied by growing cells in a semi-solid soft-agar culture medium. Single cell suspensions of NOSE plated in soft agar remained primarily as single cells and very few proliferated. They did not form colonies or display any detectable change in morphology in the first week of culture but started undergoing shrinkage between 14–21 days (Fig. 3A). In contrast, every COVCAR cell line showed distinct morphology and were alive and continued to proliferate in soft agar for approximately 50–60 days. After plating as a single cell suspension in soft agar, the C5 cell line started dividing within 24 h and by the end of the first week of culture, morphological changes such as projections or spinous processes on the surface were detected which further led to cellular interaction (Fig. 3 B, D, and F) and formation of secondary structures like acini, and tube-like structures (Fig. 3 E, G). The C5 cell line had a high propensity for colony formation and three-dimensional growth, forming a large sphere or solid ball-like structure in soft agar (Fig. 3 B, C). C6, C11, and C19 cell lines also started proliferating and forming secondary structures in soft agar similar to C5 cells but their growth rate was slower than C5 cells (data not shown). Overall, out of four cell lines studied (C5, C6, C11, C19) for soft agar culture, the C5 cell line exhibited the fastest growth rate followed by the C11 cell line, while C19 and C6 cell lines were extremely slow growing.

### COVCAR Cell Lines are Invasive in Extracellular Matrix *in vitro*

**Figure 4:**
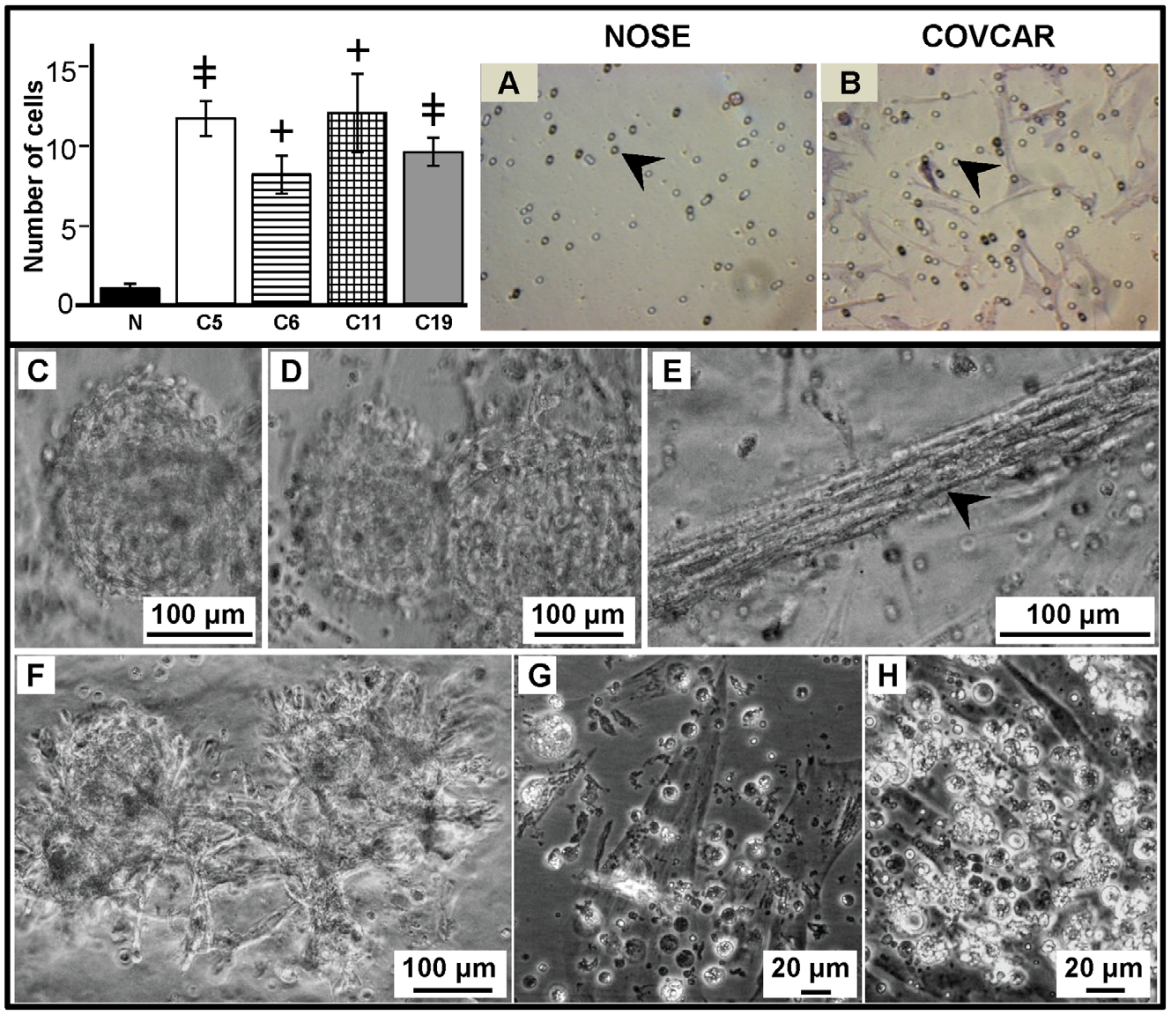
Invasion of chicken ovarian cancer (COVCAR) cells in Matrigel extracellular matrix. COVCAR cell lines (C5, C6, C11, and C19) and normal ovarian surface epithelial cells (NOSE) were layered on top of solidified Matrigel in transmembrane insert having 8 µm pores and cultured for 24 h as described in *Materials and Methods* section. The number of cells invaded through the Matrigel and pores (*arrow heads in A and B*) were counted in six fields after staining the bottom surface of the membrane. The histogram (top left) showing the number of COVCAR and NOSE (N) cells found on the bottom surface of the membrane. **A–B**. Representative photomicrographs of the membrane bottom surface showing many COVCAR cells (B) while NOSE cells were absent (A). **C–F.** Photomicrographs of COVCAR cells in Matrigel extracellular matrix. COVCAR cells formed sphere-like structures in Matrigel (C, D) that also led to outgrowth of cells (F) invading through the matrix. Stacks of COVCAR cells invading through the Matrigel matrix appeared as a rope-like structure (E). Numerous spherical COVCAR cells containing translucent vesicles (G and H) were found in the cell culture well beneath the Matrigel transmembrane insert. Many of these spherical COVCAR cells that migrated through the pores formed a layer of fibroelastic cells at the bottom of the well. Data in the histogram are represented as mean ± standard error of the mean from 3 replicates. **^+^** *P*<0.01, ^
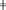
^ *P≤*0.0001 COVCAR vs NOSE.

To determine invasive properties of COVCAR cells, a Matrigel invasion assay was also performed. COVCAR cells layered on top of the Matrigel-coated chamber migrated/invaded through the extracellular matrix and through the pores of the membrane to the bottom side of the inserts within 24 h (Fig. 4 A–B). The number of COVCAR cells found on the bottom side of the membrane was significantly higher than the number of NOSE cells on this side of the membrane (Fig. 4). When the COVCAR cells were allowed to grow for longer than 24 h in Matrigel, they formed many inter-connected spheroid-like clumps (Fig. 4 C, D, and F). Groups of fibroelastic cells were arranged in linear stacks, giving an appearance of a rope-like pattern (Fig. 4 E). In all the COVCAR cell lines studied, several cells that migrated through the Matrigel layer and the pores were initially found floating in the cell culture media in the bottom well but later on attached and formed a mat of cells (Fig. 4 G–H). Such floating cells appeared spherical and contained several translucent vesicles and resembled cells when harvested from ascites (Fig. 2 A). In contrast, NOSE cells formed clumps only on the surface of Matrigel layer and only a few cells penetrated the extracellular matrix within 24 h of culture (data not shown). Taken together, our data suggest that all the COVCAR cell lines used in this study had significantly higher invasive properties than NOSE cells.

### COVCAR Cell Lines Display Wound Healing Property

**Figure 5:**
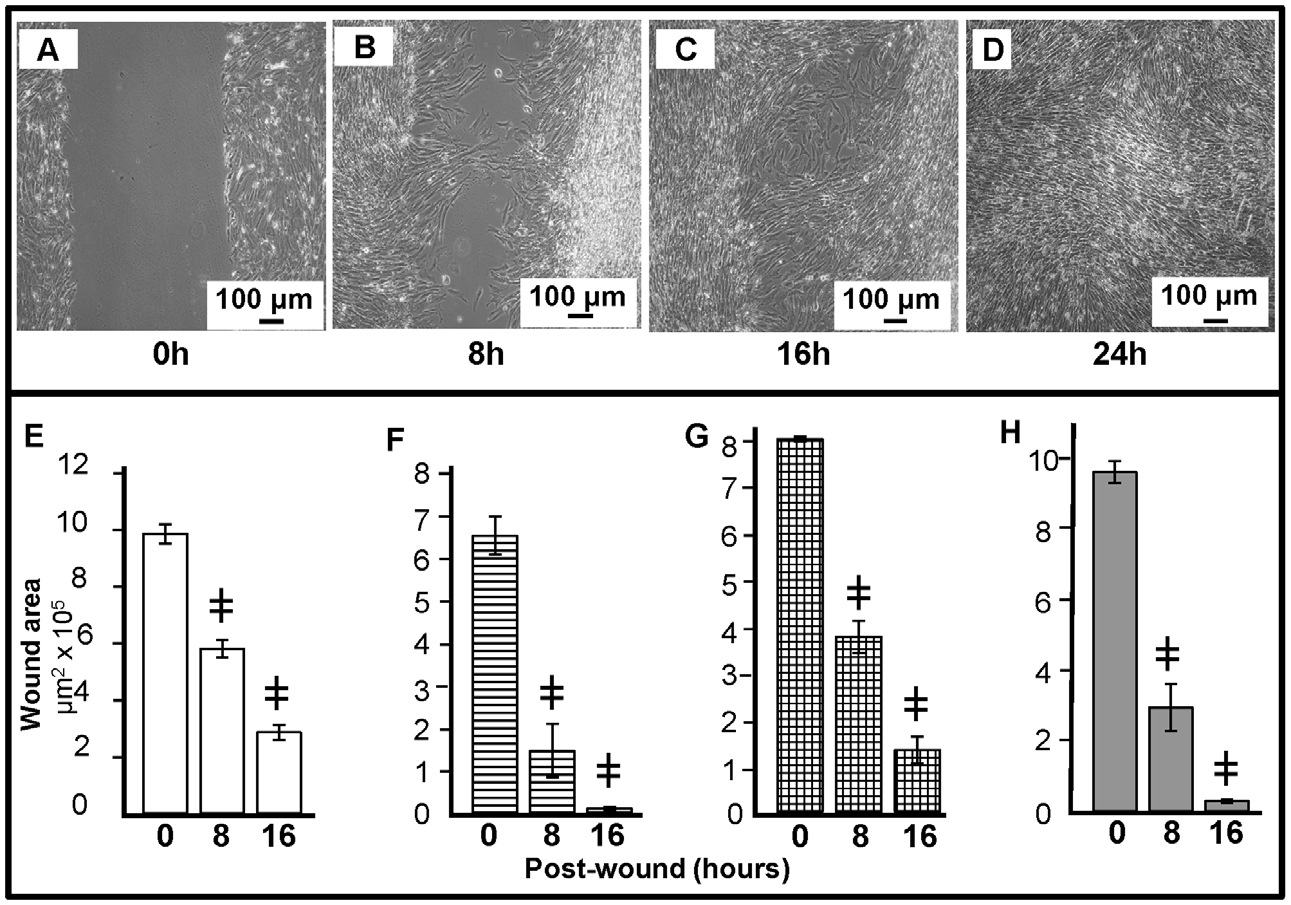
Migration and wound healing properties of chicken ovarian cancer (COVCAR) cells. A vertical wound (700–900 µm width) was created in fully-confluent COVCAR cells followed by washing in culture medium and incubation at 37°C and 5% CO_2_. Photomicrographs were taken at 0, 8, 16 and 24 h post-wound creation to determine the wound area using image analysis software. **A–D**. Representative photomicrographs of the wound at 0, 8, 16, and 24 h after creating wound in C11 cell line in 4^th^ passage. **E–H**. Wound area following 0, 8, 16, and 24 h in COVCAR cell lines C5 (E), C6 (F), C11 (G), and C19 (H). Data are represented as mean ± standard error of the mean from 3 replicates., **^
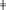
^** *P*≤0.0001 COVCAR vs NOSE.

Wound healing properties of COVCAR cell lines (C5, C6, C11, and C19) were determined by creating a wound through 100% confluent cultures. It is evident from Fig. 5 A, B, C, D that the wound (700–900 µm wide) was completely healed within 16–24 h. A significant decrease (P<0.05) in wound area was noticed 8, 16, or 24 h after creating the wound in all of the COVCAR cell lines studied (Fig. 5 E, F, G, H).

### Immunoctyochemical Evidence for Expression of Cytokeratin, E-cadherin and α-smooth Muscle Actin in COVCAR Cell Line

**Figure 6:**
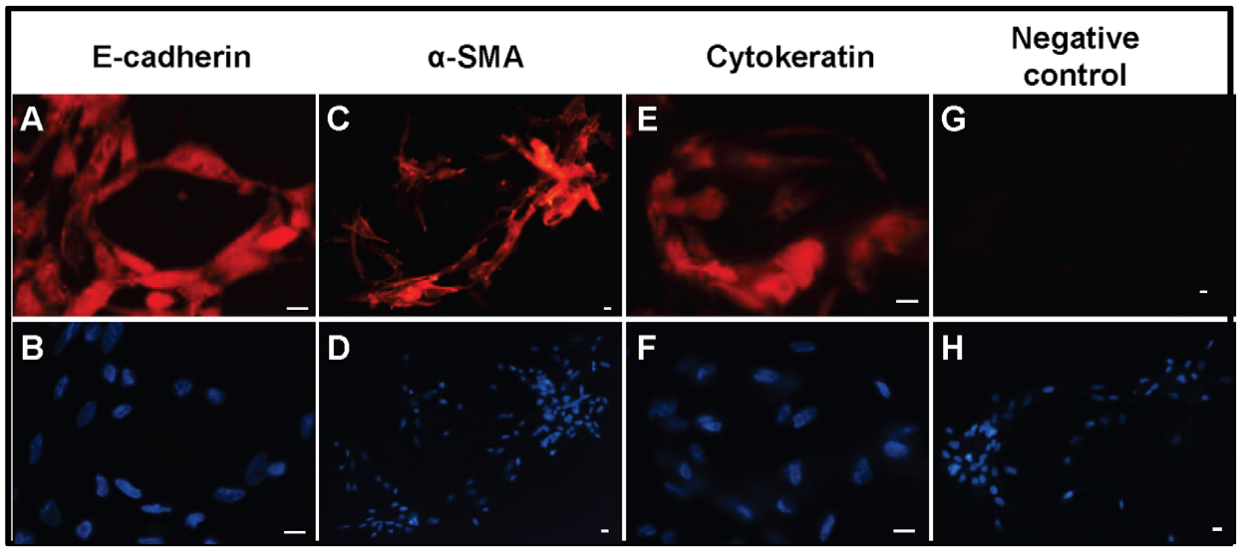
Expression of cytoskeletal proteins in chicken ovarian cancer (COVCAR) cells. Photomicrographs of chicken ovarian cancer (COVCAR) cells showing E-cadherin (A), α-smooth muscle actin (C; SMA), and cytokeratin (E) immunostaining. Paraformaldehyde-fixed COVCAR cells were immunostained as described in *Materials and Methods* section. Fixed COVCAR cells were incubated with anti-mouse IgG (G) in place of primary antibody as negative control. Nuclei were visualized with DAPI staining (B, D, F, and H) on cells immunostained with E-cadherin, SMA, or cytokeratin, respectively. Scale bars-10 µm.

COVCAR cell lines (C5, C6, C7, C11, and C19) were cultured in chamber slides and immunostained to localize E-cadherin, α-smooth muscle actin, and cytokeratin. Representative photomicrographs showing robust cytoplasmic localization of E-cadherin, α-smooth muscle actin, or cytokeratin are shown in Fig. 6 A, C, and E, respectively. Replacing the primary antibody with mouse IgG resulted in complete absence of immunostaining (Fig. 6 G).

### COVCAR Cell Lines Express Several Genes Related to Hormone Synthesis, Hormone Signaling, Cytoskeleton, and Growth Factors

**Figure 7:**
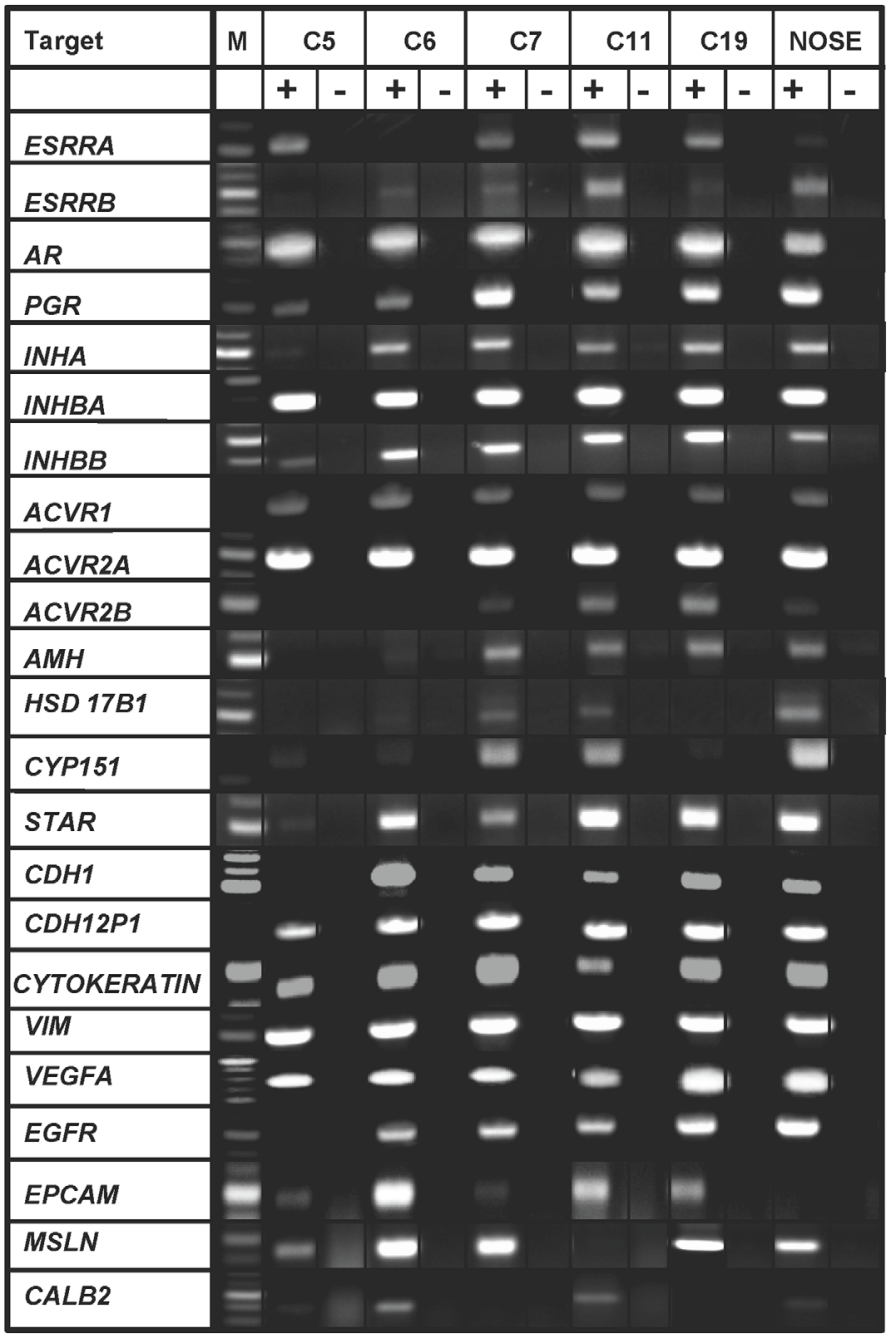
Expression of genes in chicken ovarian cancer cells (COVCAR) and normal ovarian surface epithelial cells (NOSE). RT-PCR analyses for expression of various cytoskeletal proteins, growth factors and receptors, protein/enzymes related to steroid hormone synthesis, gonadal hormone and hormone receptors in chicken ovarian cancer cell lines (C5, C6, C7, C11, C19) and normal ovarian surface epithelial cells (NOSE; n = 5 animals). Total RNA was extracted from cultured cells in passages 3–4 and treated with deoxyribonuclease-I. Approximately 250 ng of cDNA (+RT) was used as template to amplify the gene products. Contamination controls consisted of reverse transcribed RNA without reverse transcriptase (-RT). M- DNA size marker.

To characterize expression of genes related to hormone synthesis and signaling, cytoskeletal proteins, and growth factors, RT-PCR was performed using cDNA derived from selected COVCAR cell lines and NOSE cells. Fig. 7 provides representative photographs of RT-PCR products amplified for each target gene from 5 COVCAR cell lines (C5, C6, C7, C11, and C19) and representative NOSE cell cDNA while Table 2 summarizes the expression of genes in these cell lines. Most of the COVCAR cell lines (C5, C6, C7, C11, and C19) and NOSE cells studied were found to express genes related to gonadal steroidogenesis, androgen receptor (AR), progesterone receptor (PR), estrogen receptor (ER)- α, ER-β, inhibin/activin α, β_A_ and β_B_ subunits, activin receptors, anti-müllerian hormone (AMH), vascular endothelial growth factor (VEGF) and epidermal growth factor (EGF) receptor. In addition, genes encoding cytoskeletal proteins such as E-cadherin, N-cadherin, and vimentin were found to be expressed in both COVCAR cell lines and NOSE cells. Epithelial cell adhesion molecule (EpCAM) gene expression was not detectable in NOSE cells but this gene was expressed in most of the COVCAR cell lines studied (Fig. 7). Expression of mesothelin gene, a mesothelial cell marker, was found in most of the COVCAR cell lines and NOSE cells.

**Table 2:** Expression of selected gene transcripts in chicken ovarian cancer (COVCAR) cell lines and normal ovarian surface epithelial (NOSE) cells and sequences of the oligonucleotide primers used for amplification.

Gene	Accession No.	Primer Sequence	Presence	Absence
Cytoskeletal proteins				
*CDH1*	NM_001039258	fwd: 5′-tggagccgggcgagtacaatatctt-3′	C5,C6, C7, C11,C19, NOSE	
		rev: 5′-’tagtcgaacaccagcagcgagtcgta-3′		
*CDH12P1*	NM_001001615	fwd: 5′-tcagcagagcccacggagtttgtagt-3′	C5,C6, C7, C11, C19, NOSE	
		rev: 5′-’gtcccattccaaacctgatgcaagaa-3′		
*CYTOKERATIN*	XM_424502	fwd: 5′-gtgagtaccaggagctgatgaacgtcaag-3′	C5,C6, C7, C11, C19, NOSE	
		rev: 5′-ggatagacacagggagacagaggacagatg-3′		
*VIM*	NM_001048076	fwd: 5′-ctacatcacgtccagcacccgctatt-3′	C5,C6, C7, C11, C19, NOSE	
		rev: 5′-ctgcagcatctcctcctgcaacttct-3′		
*MSLN*	XM_414835	fwd: 5′-ctggccaccattgttgcagaactctt-3′	C5, C6, C7, C19, NOSE	C11
		rev: 5′-tgcattcagtgcagtggcattcaag-3′		
*EPCAM*	NM_001012564	fwd: 5′-aatggaaccacctgttggtgtgtgaa-3′	C5, C6, C7, C11, C19	NOSE
		rev: 5′-caatgacagcaataacaccagcagtca-3′		
*CALB2*	NM_205316	fwd: 5′-gagttcatggaggcatggaggaggta-3′	C5, C6, C11, NOSE	C7, C19
		rev: 5′-ggtgagctgctggatgctcatctctt-3′		
Growth factor and receptors				
*VEGFA*	AB011078	fwd: 5′-agcggaagcccaacgaagttatcaaa-3′	C5,C6, C7, C11, C19, NOSE	
		rev: 5′-gctcatgtgcgctatgtgctgactct-3′		
*EGFR*	NM_205497	fwd: 5′-ttgtggttggtctaggcatcggtctt-3′	C6, C7, C11, C19, NOSE	C5
		rev: 5′-ttgcaatctgcacacaccagttgaga-3′		
Steroid Hormone Synthesis/gonadal hormone receptors/ovalbumin				
*STAR*	AF220436	fwd: 5′-aaggagaggaagccctgcagaaatca-3′	C5,C6, C7, C11, C19, NOSE	
		rev: 5′-aggtcgatgctaagaagccacgtcaa-3′		
*HSD17B1*	NM_204837	fwd: 5′-cgcaggttcaaagtgttcgccacta-3′	C7, C11, NOSE	C5, C6,C19
		rev: 5′-caccagcgtcatgtggatgttgaag-3′		
*CYP151*	J04047	fwd: 5′-atgccatcttcacacaggctgacaaa-3′	C5,C6, C7, C19	C11
		rev: 5′-aagctcagtcccttgaagtgcttgga-3′		
*AR*	*AB193190*	fwd: 5′-aaacgactgcaccatcgacaagttca-3′	C5,C6, C7, C11, C19, NOSE	
		rev: 5′-gtgaaagatctccaacccatggcaaa-3′		
*PGR*	NM_205262	fwd: 5′-accagccagagctcccagtacagctt-3′	C5, C6, C7, C11, C19, NOSE	
		rev: 5′-tggtcagcaaggaacttggtgtttca-3′		
*ESRRA*	NM_205183	fwd: 5′-agtccaccaaggagacccggtactgt-3′	C5, C7, C11, C19, NOSE	
		rev: 5′-gcaaggttggtcaacagggtcatcat-3′		
*ESRRB*	NM_204794	fwd: 5′-gcgcagtctgcagtgactatgcttca-3′	C6, C7, C11, C19, NOSE	
		rev: 5′-ccagccaatcatgtgaaccagctctt-3′		
*FSHR*	*NM_205079*	fwd: 5′-atgtttgctttcacggtggcacttct-3′	C6, C19, NOSE	C5, C7, C11
		rev: 5′-ggcttgcatttcacagcaaccaaact-3′		
*AMH*	*U61754*	fwd: 5′-ggagacgctccctcaccaactactca-3′	C6, C7, C11, NOSE	C5, C19
		rev: 5′-gtagacggtgggcatgacgatgaagt-3′		
*INHA*	NM_001031257	fwd: 5′-agccagacaagctgctggaggaagaa-3′	C5,C6, C7, C11, C19, NOSE	
		rev: 5′-cagcccagctcctcgaaagagatgtt-3′		
*INHBA*	NM_205396	fwd: 5′-cacagcctgagatggtggaagcagta-3′	C5,C6, C7, C11, C19, NOSE	
		rev: 5′-ctgttgaaacaggcggatggtgactt-3′		
*INHBB*	NM_205206	fwd: 5′-ttggatgttcaatgtgagggctgtga-3′	C5,C6, C7, C11, C19, NOSE	
		rev: 5′-tttggttggaatgcaacaggagttca-3′		
*ACVR1*	NM_204560	fwd:5′-tgccatttctcatcgggacttgaaga-3′	C5,C6, C7, C11, C19, NOSE	
		rev: 5′-tgattcgcagggctgttagtcttgct-3′		
*ACVR2A*	NM_205367	fwd:5′-tccagtcacaccgaagccacctctat-3′	C5,C6, C7, C11, C19, NOSE	
		rev: 5′-cacagctcattccaggaaaccacgtt-3′		
*ACVR2B*	NM_204317	fwd:5′-cacgacaagggctctctcacggatta-3′	C7, C11, C19, NOSE	C5, C6
		rev: 5′-cctcaagggatgggtgctgacctatt-3′		
*SERPIN B11*	V00383	fwd: 5′-ttctcaaactgcaatggttctggttaatgc-3′		C5, C6, C7,C11, C19, NOSE
		rev: 5′-tttcctccatcttcatgcgaggtaagtaca-3′		

### Higher Levels of VEGF, N-cadherin, Vimentin, and ZEB1 mRNA in COVCAR Cell Lines

**Figure 8:**
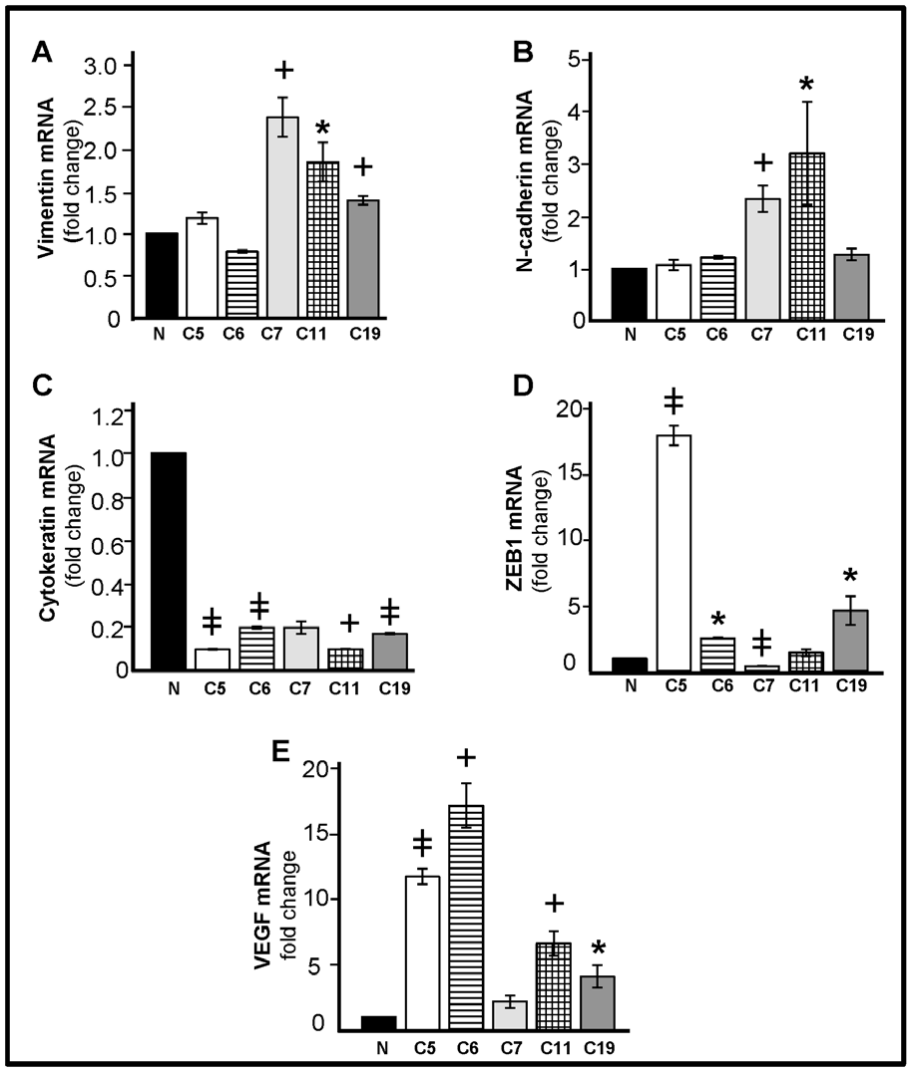
Quantification of vimentin, N-cadherin, cytokeratin, ZEB1, and VEGF mRNA in chicken ovarian cancer (COVCAR) cells. Vimentin mRNA (A), N-cadherin mRNA (B), cytokeratin mRNA (C), ZEB1 mRNA (D), and VEGF mRNA (E) abundance in normal ovarian surface epithelial cells (N; n = 5 animals) and COVCAR cell lines (C5, C6, C7, C11, C19). Total RNA was extracted from cultured cells in passages 3–4 and treated with deoxyribonuclease-I. Following reverse transcription, approximately 50 ng of cDNA was used in quantitative real-time PCR using SYBR® green as the dye to quantify vimentin mRNA, N-cadherin mRNA, cytokeratin mRNA, ZEB1 mRNA, VEGF mRNA, or β-actin mRNA in separate reactions. Each reaction was run in triplicate per cell line and the critical threshold (*C*_T_) values were subtracted from that of β-actin mRNA, averaged and converted from log-linear to linear term. *P<0.05, **^+^** *P*<0.01, ^
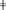
^ *P*≤0.0001 COVCAR vs NOSE; n = 3/cell line.

Quantitative real-time PCR were performed to quantify VEGF, N-cadherin, vimentin, ZEB1, and cytokeratin mRNA in COVCAR cell lines (C5, C6, C7, C11, and C19) compared to NOSE cells (Fig. 8 A, B, C, D, E). As compared with NOSE cells, vimentin mRNA levels were found to be higher (P<0.05) in C7, C11, and C19 cell lines while N-cadherin mRNA levels were higher (P<0.05) in C7 and C11 cell lines compared to NOSE cells. Cytokeratin mRNA levels were several-fold lower in C5, C6, C11, and C19 cell lines compared with NOSE cells. ZEB1 mRNA levels were higher in C5, C6, and C19 cell lines but lower in C7 cell line (P<0.05). VEGF mRNA levels were many-fold significantly higher in all the COVCAR cell lines studied compared with NOSE cells (P<0.01).

### COVCAR Cell Lines and Cancerous Ovaries Express Higher Levels of E-cadherin

**Figure 9:**
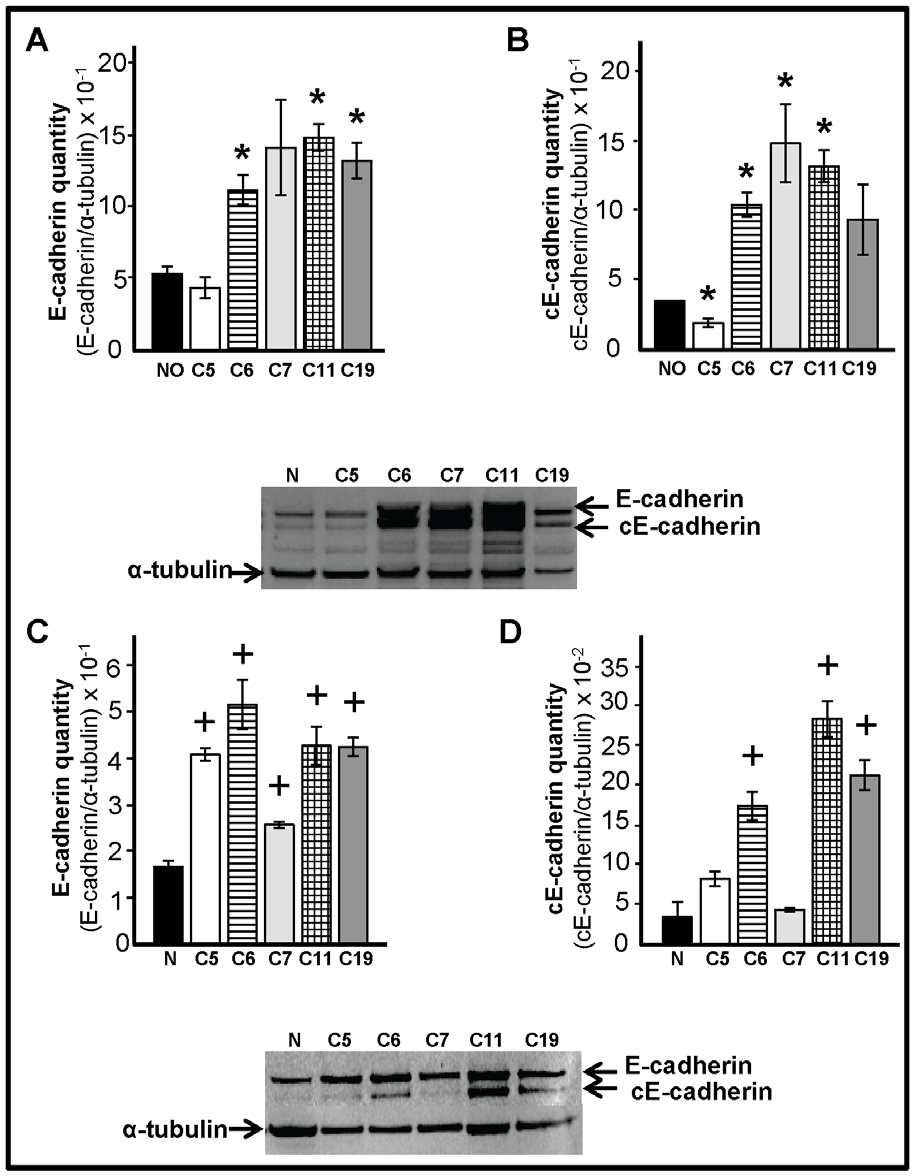
Quantification of E-cadherin in cancerous ovaries and chicken ovarian cancer (COVCAR) cells. Western blot analysis to determine the levels of E-cadherin (**A, C**) and cleavage product of E-cadherin (cE-cadherin; **B, D**) in ovaries (**A and B**) or in ovarian cancer cell lines (**C and D**). Protein extracts from cancerous ovaries or chicken ovarian cancer cell lines (COVCAR; C5, C6, C7, C11, and C19) and normal ovaries (NO; n = 5 animals) or ovarian surface epithelial cells (N; n = 5 animals) were treated with reducing agent, heat denatured, separated by electrophoresis and blotted onto PVDF membrane. E-cadherin and cE-cadherin were detected by immunostaining using mouse anti-human E-cadherin antibody. E-cadherin or cE-cadherin levels were represented as a proportion of a-tubulin levels. Data are represented as mean ± standard error of the mean from at least 3 replicates. *P<0.05, **^+^** *P*<0.01 Cancerous vs Normal or COVCAR vs NOSE.

E-cadherin levels in COVCAR cell lines, NOSE cells, cancerous and normal ovaries were determined by immunoblotting. E-cadherin levels were higher (P<0.05) in the cancerous ovaries obtained from three chickens (C6, C11 and C19) compared to normal ovaries (Fig. 9 A) while E-cadherin levels were found to be higher in all the COVCAR cell lines studied (C5, C6, C7, C11, and C19) compared to NOSE cells (Fig. 9 C). An additional band that migrates below the E-cadherin band was found in cancerous ovaries (Fig. 9 B) and in COVCAR cells lines (Fig. 9 D). This additional band, possibly representing a proteolytic cleavage product of E-cadherin, has been reported in chicken ovarian cancer lysates by other investigators [20]. Upon quantification, the levels of this E-cadherin cleavage product were found to be higher (P<0.05) in cancerous ovaries (C6, C7, and, C11; Fig. 9 B) and in some of the COVCAR cell lines (C6, C11, and C19; Fig. 9D) when compared to respective control samples. Cleavage products of E-cadherin have been found to enhance motility, invasion, survival, and proliferation of tumor cells (reviewed in [21]).

### Cancerous Ovaries Express Higher Levels of Ovalbumin but COVCAR and NOSE Cells did not Express Ovalbumin

**Figure 10:**
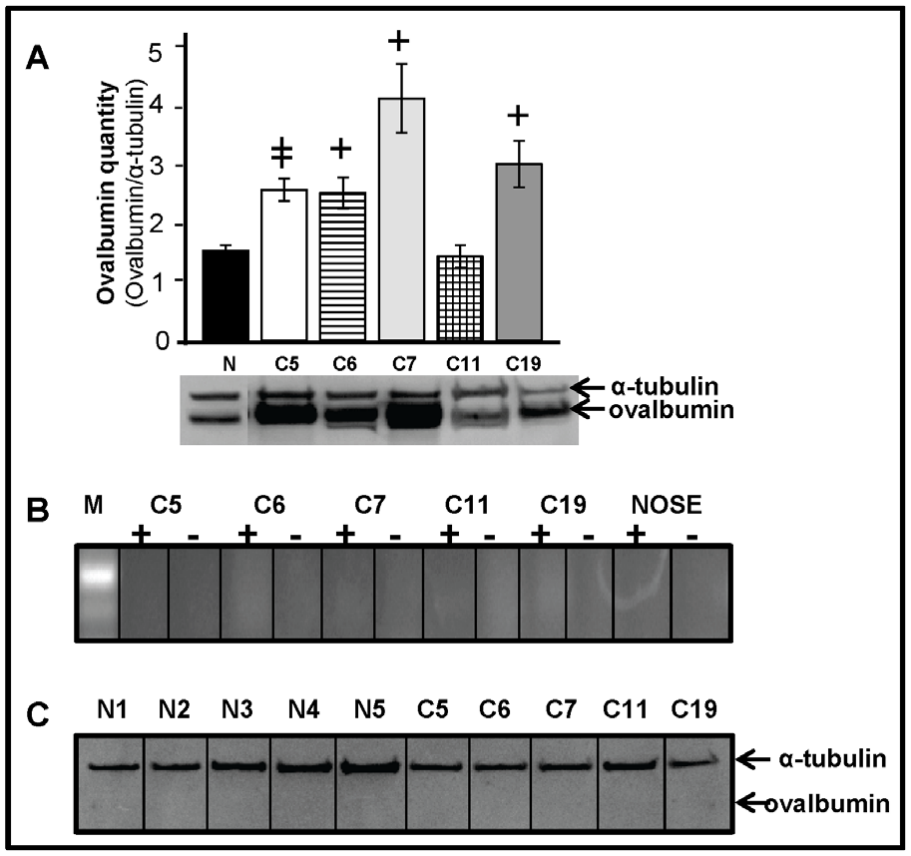
Ovalbumin expression in cancerous ovaries and chicken ovarian cancer (COVCAR) cells. A. Western blot analysis of ovalbumin levels in cancerous (C5, C6, C7, C11, C19) and normal ovaries (N; n = 5 animals) of the chicken. Protein extracts from normal and cancerous ovaries were treated with reducing agent, heat denatured, separated by electrophoresis and blotted onto PVDF membrane. Ovalbumin was detected by immunostaining using mouse anti-chicken ovalbumin antibody. Ovalbumin levels were represented as a proportion to α-tubulin levels. Data are represented as mean ± standard error of the mean from at least 3 replicates. **^+^** *P*<0.01, ^
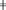
^ *P*≤0.0001 Cancerous vs Normal. **B.** RT-PCR analyses for ovalbumin mRNA expression in chicken ovarian cancer cell lines (COVCAR; C5, C6, C7, C11, and C19) and normal ovarian surface epithelial cells (NOSE). Approximately 250 ng of cDNA (+RT) prepared from COVCAR cell lines and NOSE cells (from 5 chickens) was used as template to amplify a partial ovalbumin cDNA. Contamination controls consisted of COVCAR or NOSE cell RNA untreated with reverse transcriptase (−RT). **C**. Western blot analysis to detect ovalbumin expression in COVCAR cell lines (C5, C6, C7, C11, and C19) and NOSE cells (N1, N2, N3, N4, N5). Cellular protein lysates were subjected to immunoblotting as described above to detect ovalbumin. α-tubulin immunostaining was used as positive control.

Ovalbumin levels in cancerous ovaries (C5, C6, C7, and C19) were significantly higher (P<0.01) compared to normal ovaries (Fig. 10 A). However, ovalbumin gene and protein expression were undetectable in all the COVCAR cell lines (C5, C6, C7, C11, and C19) and in all NOSE cell preparations (Fig. 10 B–C).

## Discussion

This is the first report on the characterization of malignant tumor cells derived from ascites of chickens that had developed ovarian tumor. In addition, described for the first time is a simple method for harvesting, long-term culturing, and cryopreservation of chicken ovarian tumor cells from ascites. The procedure is based on a previously reported method for culturing primary ovarian tumor cells obtained from ascites in human subjects with advanced stages of ovarian cancer [22].

All of the chickens used in this study were found to be in advanced stage (Stage III or Stage IV) of ovarian carcinoma characterized by accumulation of ascites and tumor metastasis to the peritoneum and to visceral organs. Ascites is generally composed of lymph and serous secretions from the peritoneum, fibroblasts, mesothelial cells, leukocytes, erythrocytes, exosomes, and malignant carcinoma cells [23], [24]. Ascites, however, is still considered a good source for isolation of metastatic tumor cells in single cell forms or small aggregates without the need for mechanical or enzymatic separation [22]. In our experiments, frequent exchange of cell culture media was sufficient to eliminate leukocytes, erythrocytes, and other unattached cells. MCDB/M199 culture media used in the present study was reported to discourage growth of fibroblast-like cells while allowing growth of primary OSE cells obtained from human ovary [22]. Also, the presence of epithelial and mesothelial markers such as cytokeratin, E-cadherin, and mesothelin along with several gonadal hormones and receptors confirmed the epithelial nature and ovarian origin of the cells. Many of the COVCAR cell lines were highly proliferative requiring repeated and frequent passaging before reaching senescence over a 3-month period (data not shown). All COVCAR cell lines studied exhibited anchorage-independent growth albeit each cell line had different growth and morphological characteristics. The Matrigel invasion assay revealed that the COVCAR cells were highly invasive in extracellular matrix similar to human ovarian cancer cells [25]. Short-term primary cultures of carcinoma cells obtained from human ovarian tumors, intraperitoneal tumor masses, and ascites were found to secrete a variety of extracellular matrix degrading proteinases [26]. In this regard, chicken ovarian tumors were found to overexpress matrix metalloproteinase 3 gene [27]. Collectively, it is plausible that the COVCAR cell lines secrete extracellular matrix-degrading enzymes to render them invasive. Some of the COVCAR cell lines displayed robust growth in soft agar by forming numerous colonies. COVCAR cells in some of the colonies tended to form acini and tube-like structures similar to vascular mimicry described for human ovarian cancer cell lines [28]. Taken together, our data suggest that COVCAR cells obtained from ascites display several morphological characteristics and invasiveness similar to human ovarian epithelial tumor cells.

In the present study, COVCAR cell lines and NOSE cells were found to express several genes related to steroid hormone synthesis, gonadal hormone receptors, cytoskeleton, growth factor and growth factor receptors. All of the five COVCAR cell lines studied and NOSE cells expressed the N-cadherin gene and E-cadherin protein. In humans, N- cadherin is reported to be expressed in both human normal OSE and immortalized ovarian cancer cell lines (reviewed in [29]). In addition to chicken, E-cadherin appears to be expressed in the normal OSE of rhesus macaque [30], rats [31], and humans (columnar cells only; [32]). We found that all of the COVCAR cell lines and NOSE cells expressed cytokeratin and vimentin as in human cancer cell lines and OSE cells [29], [33]. In support of our data, cytokeratin was found to be expressed in cultures of normal OSE cells isolated from chicken ovarian explants at least in the first three days of cultures while weak expression of vimentin was noticed only after 10 days of culture [16]. Our immunocytostaining data also support RT-PCR expression of cytokeratin and E-cadherin in COVCAR cell lines. Interestingly, we found that COVCAR cells express α-smooth muscle actin that is typically found in human epithelial ovarian cancer cells to facilitate cellular motility [34]. Consistent with the origin of the OSE, all COVCAR cell lines and NOSE cells were found to express mesothelin, a mesothelium-specific protein. Epithelial cell adhesion molecule (EpCAM) was found to be expressed in all COVCAR cell lines studied but not in NOSE cells suggesting the metastatic nature of the COVCAR cells [35] and the use of EpCAM as a potential ovarian tumor marker.

We found that most of the COVCAR cell lines studied and NOSE cells express enzymes/proteins essential for gonadal steroidogenesis and receptors to gonadal hormones suggesting that COVCAR and NOSE cells could synthesize and respond to estrogen, progesterone, and testosterone. This is consistent with expression of AR, PR, ERα, and ERβ in human ovarian cancer cell lines and normal OSE [36], [37]. In a previous study, cultured normal OSE cells responded to estradiol treatment by increasing the levels of PR [16]. In addition, both COVCAR cell lines and NOSE cells expressed inhibin/activin α, β_A_ and β_B_ subunits and activin receptors as reported in human OSE and epithelial ovarian cancer cell lines [38]. This is the first report on expression of anti-müllerian hormone (AMH) in NOSE cells and in three out of the five COVCAR cell lines studied, possibly suggesting a hitherto unknown role of AMH in OSE cells and/or perhaps its absence in certain types of ovarian neoplasms. AMH expression in NOSE cells is intriguing as AMH is typically expressed by granulosa cells in the chicken ovary [39] and to a lesser extent in liver [40]. A contamination of our NOSE cell preparation with granulosa cells is unlikely as gentle scratching of the follicular surface to release the surface epithelium would not reach the granulosa cell layer located beneath several layers of thecal cells.

Our qPCR data suggest that VEGF mRNA expression is significantly higher in COVCAR cells compared with NOSE cells. This is consistent with higher VEGF mRNA expression levels reported in ascites-derived cells compared to cancerous or normal ovaries of the chicken [19]. VEGF is a critical factor involved in angiogenesis [41] and facilitates ascites development and ovarian cancer cell metastasis [42]. Therefore, higher VEGF mRNA levels in COVCAR cell lines may be suggestive of the metastatic nature of the ovarian tumor cells. Advanced stages of ovarian cancer involve a mechanism called epithelial mesenchymal transition (EMT) in which epithelial cancer cells lose cytokeratin expression but gain mesenchymal markers such as N-cadherin and vimentin [43]–[45] to facilitate metastasis. Not surprisingly, our data also suggest that the levels of N-cadherin and vimentin mRNA are higher with the concomitantly lower cytokeratin mRNA levels in some of the COVCAR cell lines compared to NOSE cells suggestive of EMT and metastasis. Furthermore, zinc finger E-box binding transcription factor (ZEB1) is reported to enhance EMT by repressing the expression of epithelial phenotypic genes and activating the expression of genes associated with a mesenchymal phenotype [46]–[48]. The mesenchymal appearance of COVCAR cell lines and higher levels of ZEB1 mRNA in C5, C6, and C19 cell lines further strengthen our conclusion that these ascites-derived cells are undergoing EMT.

Our Western blotting data (Fig. 9) suggests that levels of E-cadherin are significantly higher in cancerous ovaries compared to normal ovaries. This is consistent with a previous report that documented greater E-cadherin mRNA and protein expression in cancerous ovarian tissue lysates, and intense E-cadherin expression localized to the adenocarcinomatous cells in chicken ovarian cancer tissues [20]. In addition, two independent studies reported 2-, or 40-fold higher expression of E-cadherin mRNA in chicken ovarian tumors compared to normal ovaries using microarray gene expression analysis [49], [50]. Consistent with greater E-cadherin expression in ovaries as shown by us and others, all of the COVCAR cells lines studied showed greater E-cadherin expression compared to NOSE cells, further supporting the origin and malignant nature of the COVCAR cells. Human ascites-derived primary ovarian tumor cells and immortalized ovarian tumor cell lines such as OVCAR-3 were found to express E-cadherin [51]. Although E-cadherin is an adherent membrane protein responsible for maintaining intercellular junctions in highly differentiated epithelial cells [52], [53], it is also implicated in promoting proliferation of ovarian tumor cells by activating Akt/MAPK signaling pathways [54]. Therefore, higher levels of E-cadherin in metastatic COVCAR cells may possibly influence cellular proliferation.

In the present study, ovalbumin levels were found to be higher in most cancerous ovaries compared to normal ovaries. This is consistent with previous reports on higher ovalbumin gene expression [49] and intense immunostaining of ovalbumin [55] in cancerous chicken ovarian tissues. In contrast, none of the COVCAR cell lines or NOSE cells was found to express ovalbumin gene or protein in the present study. Ovalbumin is a major oviductal protein and its absence in COVCAR cell lines may indicate that these cells did not originate from the oviduct. Alternatively, ovalbumin expression may have been suppressed in metastatic cells due to insufficient or lack of estrogen, a critical factor augmenting ovalbumin expression.

Taken together, our data suggest that the ascites derived COVCAR cells most likely arise from cancerous ovaries and display both epithelial and mesenchymal characteristics as well as anchorage-independent growth and invasiveness. At present, COVCAR cell lines represent the only cell lines isolated from adult chickens without the use of carcinogens or genetic manipulation and are likely to advance our knowledge on the cellular and molecular biology of ovarian tumor cells. Future studies should focus on determining mutations in tumor-associated genes in COVCAR cells and to characterize gene networks to elucidate changes in cellular pathways.

## Materials and Methods

### Animals

Fifty Single-comb White Leghorn female (3–4 years old) chickens from the Pennsylvania State University flock were maintained in individual cages at the Poultry Education and Research Center of the Pennsylvania State University and exposed to a daily photoperiod of 16 h light and 8 h dark. A commercial laying hen mash diet and water were provided for ad libitum consumption. Egg production was recorded daily to determine ovulatory cycle patterns over extended periods of time. All animal procedures were carried out in accordance with the recommendations contained in the Guide for the Care and Use of Laboratory Animals of the National Institutes of Health. This study and the animal procedures adopted were approved by the Institutional Animal Care and Use Committee of the Pennsylvania State University (protocol# 32834).

### Tissue Collection and Culture of Ascites-derived Ovarian Tumor Cells

Seventeen of the 50 chickens which exhibited anovulation for 1 month were selected and euthanized by decapitation. The abdomen was accessed aseptically to examine all the visceral organs including ovary and oviduct for the presence of tumor mass. Ascites, if any, were collected and visceral organs (ovary, oviduct, and intestines) were excised and stored in liquid nitrogen until used for protein and RNA extraction. A part of the ovary was fixed in Bouin fixative solution for histology. To serve as a control, tissues from age-matched healthy and regularly ovulating chickens were also collected as above. Bouin’s solution-fixed ovary tissue was processed and embedded in paraffin at the Pennsylvania State University Microscopy Core facility. Four-µm thick ovary tissue slices were prepared and stained with hematoxylin and eosin for examination by a board-certified veterinary pathologist (TKC). Cells present in ascites collected from each animal were isolated by centrifugation at 125×g for 5 min at 25°C and plated into gelatinized T-75 flasks (BD Biosciences, Bedford, MA) in growth medium (GM) that consisted of 1∶1 MCDB105:M199 culture medium (Sigma-Aldrich, St. Louis, MO), 10% chicken serum, 5% horse serum, 5% fetal bovine serum (FBS; Sigma-Aldrich), gelatin (Fisher Scientific, Pittsburgh, PA), MEM amino acid, L-alanine-L-glutamine dipeptide (Mediatech, Manassas, VA), glucose (Invitrogen, Carlsbad, CA), and sodium pyruvate (Sigma-Aldrich). In addition, penicillin-streptomycin-fungizone antimicrobial solution (Invitrogen) was supplemented to the GM when growing ascites-derived chicken ovarian cancer cells (COVCAR) during the initial first passage. The flasks containing COVCAR cells were incubated at 37°C and 5% CO_2_ and the culture media was changed every 48–72 h to promote cellular growth. COVCAR cells were passaged every 4–7 days by first detaching them with TrypLE (Invitrogen) and replating at a 1∶1 to 1∶4 ratio in GM. Several batches of COVCAR cells from various passages were frozen in GM containing 10% dimethyl sulfoxide (Sigma-Aldrich) and stored in liquid nitrogen vapor. The viability of frozen COVCAR cells of different passages was tested by thawing frozen COVCAR cells and plating them in GM.

To serve as control for COVCAR cells, normal ovarian surface epithelial (NOSE) cells from pre-ovulatory follicles of regularly ovulating Leghorn chickens (n = 5) were utilized. Chickens were euthanized by decapitation and ovaries containing follicles were aseptically removed. The follicles were first rinsed in sterile phosphate buffered saline (PBS) and after removing the fibro-vascular layers, the follicles were immersed in 1X TrypLE (Invitrogen) at 37°C for 25–30 min to loosen the surface epithelial cells. The follicles were gently scraped using a human cytobrush (Cooper Surgical, Trumbull, CT) to release the surface epithelial cells into GM. Cells were then sedimented by centrifugation at 125×g, 4°C for 5 min and the resulting cell pellet was reconstituted in ice-cold MEM. The cells were plated in sterile gelatinized T-75 tissue culture flasks (BD Biosciences), incubated at 37°C under 5% CO_2,_ and allowed to reach 80–90% confluence before extraction of cellular RNA and protein.

### Immunoflourescent Detection of Cytoskeletal Proteins in COVCAR Cells

To localize selected epithelial markers and cytoskeletal proteins, COVCAR cells (150,000) were cultured in Lab-Tek glass chamber slides (Thermo Scientific, Rochester, NY) at 37°C, 5% CO_2_. After 48 h of culture, cells were washed in cold PBS, fixed in 2% paraformaldehyde and stored in PBS at 4°C until the immunostaining procedure was performed. The slides were washed in Tris-buffered saline (TBS) containing 0.5% Triton X-100 (TBSX; Sigma-Aldrich), and then incubated in 1% goat serum (Vector Laboratories, Burlingame, CA) in TBSX for 1 h at ambient room temperature followed by overnight incubation in respective primary antibodies [anti-α-smooth muscle actin (5 µg/ml; Sigma-Aldrich;), anti-cytokeratin AE1/AE3 (2 µg/ml; Santa Cruz Biotechnology, Santa Cruz, CA), anti-E-cadherin (5 µg/ml; BD Biosciences, San Jose, CA)] at 4°C. After several washes and brief incubation in 1% goat serum, slides were incubated in goat anti-mouse IgG conjugated to Alexa-546 (10 µg/ml; Invitrogen) for 1 h at ambient room temperature. After several washes in dark, ProLong Gold antifade reagent containing DAPI (Invitrogen) was applied and slides were coverslipped for visualization in an Axioskop microscope (Zeiss, New York, NY) and photomicrographed using Axiocam videocamera (Zeiss).

### Soft Agar Colony Formation Assay

Anchorage-independent growth was monitored by the soft agar colony formation assay in 60×15 mm cell culture petri dishes. TrypLE (Invitrogen)-dissociated COVCAR and NOSE cells (500,000 cells per dish) were suspended in 3 ml RPMI 1640 medium containing 10% FBS and 0.35% DNA grade agar (Invitrogen) at 40°C and poured on top of solidified RPMI 1640 containing 10% FBS and 0.5% DNA grade agar. The agar plates with cells were incubated at 37°C under 5% CO_2_ for 2–3 weeks in a humidified incubator. Colony formation and morphology of COVCAR and NOSE cells were assessed using phase contrast microscopy.

### In vitro Invasion Assay

The upper chamber of transmembrane cell culture inserts (BD Biosciences) having 8 µm size pores was coated with 200 µl Matrigel basement membrane matrix (BD Biosciences; Lot#32489) diluted (2 mg/ml) in serum-free 1X RPMI media and allowed to solidify by incubation at 37°C, 5% CO_2_ for 2 h. COVCAR (passages 3–5) and NOSE (passages 1–3) cells were dissociated using TrypLE and approximately 200,000 cells in serum-free 1X RPMI media were layered on the Matrigel-coated inserts. The inserts were suspended in 24-well cell culture plates containing 750 µl 1X RPMI medium containing 10% FBS and incubated at 37°C, 5% CO_2_ for 24 h. Un-invaded cells in the Matrigel layer on the upper surface of inserts were removed using a cotton swab and cells that invaded through the Matrigel and 8 µm pores to the lower surface of the inserts were fixed in methanol and stained with Giemsa stain. The lower surface of the insert was photographed (320×magnification) and the number of invaded cells were counted and averaged from six non-overlapping fields.

### Wound Healing Assay

COVCAR cell lines (n = 4) were grown in gelatinized 6-well plates (3 wells/cell line) using GM as described above. When the cell growth reached approximately 100% confluence, a vertical wound (700–900 µm width) was created using a sterile pipette tip followed by a washing in GM and incubation at 37°C and 5% CO_2_. Photomicrographs were taken at 0, 8, 16 and 24 h post-wound creation to determine the area of the wound/wound healing using Axiovison image analysis software 4.7.1 (Zeiss).

### RNA Extraction and Reverse Transcription PCR (RT-PCR)

Total RNA was extracted from COVCAR cell lines (C5, C6, C7, C11, and C19; passages 3–4) and NOSE cells (n = 5 animals; passages 2–3) using RNeasy kit (QIAGEN, Valencia, CA) following the manufacturer’s instructions. Briefly, the cells were washed when they reached 80–90% confluence with cold PBS and a cell lysate was made in lysis buffer containing β-mercaptoethanol. Cell lysates were stored at −80°C until processed. Total RNA was treated with DNAse-I (Qiagen) on RNeasy column to digest genomic DNA. First strand cDNA was prepared by reverse transcription using 1 µg of total RNA, random primer 12 (New England Biolabs, Beverly, MA), Moloney murine leukemia virus (M-MuLV) reverse transcriptase (New England Biolabs), RNaseOUT (Invitrogen), and dNTP mix (Roche Applied Sciences, Indianapolis, IN) in a 20 µL reaction (+RT). To determine expression of various genes in COVCAR and NOSE cell lines, a touchdown PCR was performed using 50 ng cDNA, 300 nM dNTP (Roche Biochemicals), Taq Polymerase (New England Biolabs) and 300 nM forward and reverse primers (Table 1) using the following thermocycle: 94°C for 1 min, 30 cycles of 94°C for 5 sec, and 72–68°C for 3 min. Annealing and primer extension were done at 72, 70, and 68°C during 1–5, 6–10, and 11–30 cycles, respectively. For negative controls, reverse transcription reactions using 1 µg total RNA from COVCAR and NOSE cell lines that contained water in place of reverse transcriptase (-RT) were used as a template. PCR products were separated by electrophoresis, stained with ethidium bromide and visualized using ultraviolet light.

### Real-time Quantitative PCR

To determine whether the levels of cytokeratin, N-cadherin, vimentin, ZEB1 and VEGF mRNA differed in COVCAR and NOSE cell lines, a real-time quantitative PCR (qPCR) was performed as described previously [56]. Total RNA was extracted from COVCAR and NOSE cell lines and reverse transcribed as described above. Briefly, 50 ng of cDNA prepared from total RNA extracted from COVCAR and NOSE cell lines was mixed with 1X PerfeCTa SYBR Green Fastmix (Quanta Biosciences, Gaithersburg, MD), and 300 nM forward and reverse primers (Table 3). Reactions were carried out in 7500 Fast-Real Time PCR System (Life Technologies) with the following thermocycle: 95°C for 20 sec followed by 35 cycles of 95°C for 3 sec, 55°C for 10 sec and 63°C for 30 sec. At the end of amplification, a melting curve analysis was done by heating the PCR products from 60°C to 95°C, held for 15 sec in increments of 0.2°C, and the fluorescence detected to confirm the presence of a single amplification product. Samples from each cell line were run in triplicate to obtain average C_T_ values for target mRNA and β-actin. The log-linear threshold values (C_T_) during the exponential phase of the PCR for target mRNA were subtracted from that of β-actin mRNA. Target mRNA quantity was expressed as a proportion to β-actin quantity following 2^–ΔΔC^_T_ method for converting log-linear C_T_ values to linear term [57] and analyzed.

**Table 3:** Sequences of the oligonucleotide primers used for amplification and quantification of selected gene transcripts in chicken ovarian cancer (COVCAR) cell lines and normal ovarian surface epithelial (NOSE) cells.

Gene	Accession No.	Primer Sequence
*cdh12p1*	NM_001001615	fwd: 5′-actggtgacattattaccgtagca-3′
		rev: 5′-tagccactatgacatccactctgt-3′
*Cytokeratin*	XM_424502	fwd: 5′-gcgacctacaggaagctgct-3′
		rev: 5′-cccaaatcctcctgagtaccc-3′
*VIM*	NM_001048076	fwd: 5′-gaagctgctaactaccaggacact-3′
		rev: 5-taggcatgttaatcctgctctctt-3′
*ZEB1*	NM_205131	fwd: 5′-tcttacggtgaagtctgagaaagata-3′
		rev: 5-agtgctttaattcttgaagtgcatta-3′
*VEGFA*	AB011078	fwd: 5′-cacatgagcttcttacagcacagt-3′
		rev: 5′-acaaacaagtgctttctcctctct-3′
*ACTB*	NM_205518	fwd: 5′-ctggcacctagcacaatgaa-3′
		rev: 5′-ctgcttgctgatccacatct-3′

### Protein Extraction and Immunoblotting Analysis

Protein extracts of normal and cancerous ovarian samples (n = 5) and COVCAR cell lines (C5, C6, C7, C11, and C19; passages 3–4) and NOSE cells (n = 5; passages 2–3) were prepared as described previously [56], [58]. Briefly, ovary tissue devoid of preovulatory follicles were homogenized in RIPA lysis buffer [10 mM Tris-HCl, 150 mM NaCl (pH 8.0, 1% Nonidet P-40, 0.5% sodium deoxycholate, 0.1% SDS] containing protease inhibitor and phosphatase inhibitor cocktail (Sigma Aldrich) to prepared lysates. Similarly, COVCAR and NOSE cell cultures, at 80–90% confluence, were washed in cold PBS and cellular lysates prepared in RIPA lysis buffer. Total protein concentration was estimated by a protein dye-binding assay using a commercial kit (Bio-Rad) with chicken ovalbumin as the standard. Aliquots of protein extracts were stored at −80°C until analyzed by Western blotting. One-dimensional gel electrophoresis was performed under reducing conditions using the NuPAGE Novex Bis-Tris minigel system (XCell SureLock Minicell; Invitrogen) according to manufacturer’s recommendations as described previously [56]. Briefly, 15–30 µg protein extract was denatured using NuPAGE reducing agent and heated for 10 min at 70°–100°C and separated on 10% Bis-Tris polyacrylamide gel (Invitrogen) and 3-(N-morpholino)propanesulfonic acid (MOPS) running buffer under reducing conditions. Proteins from the gel were electro-transferred onto Immun-Blot polyvinylidene difluoride (PVDF) membranes (0.2 µm; Bio-Rad). Membranes were incubated in blocking buffer (5% non fat dry milk; Cell Signaling Technology, Boston, MA) for 2 h at room temperature. Membranes were then incubated with affinity-purified mouse anti-human E-cadherin antibody (0.05 µg/ml; BD Biosciences) or mouse anti-chicken ovalbumin antibody (1 µg/ml; Sigma-Aldrich) in blocking buffer overnight at 4°C with gentle agitation. After several washes, membranes were incubated in horseradish peroxidase labeled goat anti-mouse IgG (0.08 µg/ml; Pierce, Rockford, IL) in blocking buffer for 1 h at room temperature with gentle agitation. Membranes were then treated with ECLPlus Chemiluminescence Detection Reagent (Amersham Biosciences, Piscataway, NJ) as per manufacturer’s recommendations. Chemiluminescent signals were detected using the Storm 860 Optical scanner (Amersham Biosciences) and analyzed using Image Quant TL software (Amersham Biosciences). Membranes were reprobed with mouse anti-human α-tubulin antibody (0.7 µg/ml; Sigma-Aldrich) followed by incubation in horseradish peroxidase labeled goat anti-mouse IgG (Pierce; 0.08 µg/ml) in blocking buffer. The chemiluminescent signal was then detected as described previously. E-cadherin and ovalbumin protein quantity was expressed as a proportion of α-tubulin levels and compared between cancerous and normal ovaries or between COVCAR cell lines and NOSE cells.

### Statistical Analyses

All data analyses were conducted using Statistical Analysis System (SAS Institute, Cary, NC, USA). Levels of E-cadherin, N-cadherin, ovalbumin, ZEB1 and vimentin protein or mRNA and invasiveness of COVCAR cell lines were compared with that of NOSE cell lines using student’s *t*-tests. Area of wound at 8, 16, or 24 h were compared to 0 h for each COVCAR cell line using student’s *t*-tests. A probability level of P≤0.05 was considered statistically significant.
